# Degenerative Jargon Aphasia: Unusual Progression of Logopenic/Phonological Progressive Aphasia?

**DOI:** 10.3233/BEN-2012-110218

**Published:** 2013

**Authors:** Paolo Caffarra, Simona Gardini, Stefano Cappa, Francesca Dieci, Letizia Concari, Federica Barocco, Caterina Ghetti, Livia Ruffini, Guido Dalla Rosa Prati

**Affiliations:** ^1^Department of NeuroscienceUniversity of ParmaParmaItaly; ^2^Outpatient Clinic for the Diagnosis and Therapy of Cognitive DisordersAUSLParmaItaly; ^3^Department of Clinical NeuroscienceVita-Salute San Raffaele HospitalMilanItaly; ^4^Medical Physic DepartmentAzienda Ospedaliero-UniversitariaParmaItaly; ^5^Department of Nuclear MedicineAzienda Ospedaliero-UniversitariaParmaItaly; ^6^Poliambulatorio Dalla Rosa PratiCentro Diagnostico EuropeoParmaItaly

**Keywords:** Primary progressive aphasia, logopenic aphasia, language, jargon

## Abstract

Primary progressive aphasia (PPA) corresponds to the gradual degeneration of language which can occur as nonfluent/agrammatic PPA, semantic variant PPA or logopenic variant PPA. We describe the clinical evolution of a patient with PPA presenting jargon aphasia as a late feature. At the onset of the disease (ten years ago) the patient showed anomia and executive deficits, followed later on by phonemic paraphasias and neologisms, deficits in verbal short-term memory, naming, verbal and semantic fluency. At recent follow-up the patient developed an unintelligible jargon with both semantic and neologistic errors, as well as with severe deficit of comprehension which precluded any further neuropsychological assessment. Compared to healthy controls, FDG-PET showed a hypometabolism in the left angular and middle temporal gyri, precuneus, caudate, posterior cingulate, middle frontal gyrus, and bilaterally in the superior temporal and inferior frontal gyri. The clinical and neuroimaging profile seems to support the hypothesis that the patient developed a late feature of logopenic variant PPA characterized by jargonaphasia and associated with superior temporal and parietal dysfunction.

## 1. Introduction

Progressive language disorders without generalized dementia are defined primary progressive aphasia (PPA) [1]. PPA is usually associated with the degeneration of the language dominant network and the dysfunctional pattern shown with neuroimaging includes mainly left fronto-temporal and parietal regions [2].

Patients with PPA are commonly classified as having nonfluent or fluent speech production. Nonfluent speech output or nonfluent/agrammatic PPA may include or not apraxia of speech, while fluent output corresponds to semantic variant PPA or logopenic variant PPA [3]. The nonfluent/agrammatic PPA clinical picture corresponds to a deficit mainly in left frontal and insular regions. Semantic variant PPA presents with fluent grammatically correct speech, single words comprehension deficit and difficulty in objects meaning

ISSN 0953-4180/13/$27.50 © 2013 – IOS Press and the authors. All rights reserved recognition, caused by atrophy in bilateral anterior temporal regions. Logopenic variant PPA shows phonemic paraphasia, word-finding hesitation, slow speech and decreased verbal output with long word-finding pauses and preserved grammar and articulation, corresponding to left posterior temporal and parietal lobules degeneration. The inferior parietal lobule would be the site for phonological store portion of the phonological loop [4,5].

Patients with permanent and spontaneous jargon language appear to be more common in acute neurological disorders involving critical language networks, in particular Wernicke's aphasia, than in neurodegenerative disease. There are two case reports of jargonagraphia, a condition often, but not invariably, associated with jargonaphasia. LEA was a patient with severely reduced speech production and defective articulation, affected by what seems to be a severe, rapidly progressive form of fronto-temporal degeneration [6], who produced fluent, neologistic writte jargon. FM, a patient described by the Cambridge group in at least seven papers, appears to be a case of the semantic variant of PPA. He never developed a spoken jargon. After 7 years of follow up he produced fluent, "empty" speech with perseveration of a set of "general" words (special, set, etc.). In contrast, his written production was fluent, replete of written neologisms that followed the graphotactic rules of English [7]. Two patients with fluent jargonaphasia were present among the pathological series reported by Deramecourt and colleagues [8]. Both showed left temporo-parietal hypoperfusion on SPECT, and had a pathological diagnosis of Alzheimer's disease. Two detailed case reports of neologistic jargon in PPA have been published by Rohrer and colleagues [9]. The first case presented with word-finding difficulties, which progressed to neologistic jargonaphasia associated with semantic impairment. The imaging data were atypical for the semantic variant of PPA, as they showed progressive atrophy of the temporal lobes, without an anterior-posterior gradient, and of the parietal cortex. The second presented with non-fluent, agrammatic production, but in the next year started to produce profuse neologisms in speaking and reading. Auditory comprehension also deteriorated. Imaging finding revealed progressive perisylvian atrophy, with severe involvement of the inferior parietal lobule.

The present single case study aimed to describe the clinical evolution of a similar patient who developed neologistic jargon in the course of PPA, reporting both cognitive and neuroimaging correlates of this unusual clinical picture.

## 2. Case study

The patient is a 68-year-old right handed woman with 18 years of education, teacher of Italian literature, who, ten years ago, came to our observation for language impairment.

At the onset of the disease in 2001, the patient showed word-finding difficulties with prolonged anomic pauses. In addition, neuropsychological testing disclosed mild executive dysfunction, whereas, language comprehension, syntax, semantic, memory, praxic and visuo-perceptual abilities were in the normal range (see Table 1).

CT scan revealed mild frontal atrophy. Tc-HMPAO SPECT disclosed a reduced regional cerebral blood flow (rCBF) in right inferior frontal areas and in left temporo-parietal and occipital regions.

Cognitive follow-ups showed a worsening of linguistic abilities, due to the progressive occurrence of phonemic paraphasias and appearance of neologisms. Deficits in repetition, objects naming, verbal short-term memory, verbal learning, selective attention, phonemic and semantic fluencies were also observed. She had a stressful response to illness, with depressive symptoms and apathy, caused by a complete insight of disease.

After January 2004, due to the lack of family compliance, the patient's follow-up was interrupted. We have no additional clinical data until 2008. At this time, a neuropsychological assessment, cerebral MRI, $^{18}$F-FDG PET, cerebrospinal fluid (CSF) analysis and APOE genotype survey were carried out. The cognitive assessment showed a severe impairment of speech production and repetition, characterized by a fluent but totally unintelligible language. Verbal comprehension, reading and writing were completely lost, while prosody and communicative gestures continued to be relatively appropriate to the context. The severe language impairment prevented any further neuropsychological assessment. Along with the language impairment, the patient developed a loss in daily functions (sparing eating and body movement), while depression and apathy were no longer present. The neurological examination was unremarkable. She developed stereotyped behaviour with the tendency to compulsive wandering and hoarding (pathological collection of objects, such as leaves or stones). Emotional response to both unpleasant (see images of dead at the television) and pleasant (see a baby) stimuli was preserved.

Some examples of her speech in different conversational situations are reported below. During the clinical interview she spontaneously manifested a jargon

Table 1
Neuropsychological testing of the patient in the disease course

| Neuropsychological test | Cut-off normal range | May 2001 | May 2002 | July 2003 | January 2004 | October 2008 |
|---|---|---|---|---|---|---|
| Mini Mental State Examination (MMSE) | ⩾ 23.8 | 26 | 29 | 27 | 18 | N.A. |
| Activity Daily Living (ADL) | | 6/6 | 6/6 | 6/6 | 6/6 | 2/6 |
| Instrumental Activities of Daily Living (IADL) | | 8/8 | 8/8 | 7/8 | 7/8 | 0/8 |
| Mental Deterioration Battery (MDB) | | | | | | |
| Progressive Matrices (PM 47) | ⩾ 18.96 | 26.2 | 24.45 | 32.55 | 28.45 | N.A. |
| Verbal Fluency | ⩾ 17.35 | 20.2 | 9.5* | 9.5* | 6.5* | N.A. |
| Sentence Construction | ⩾ 8.72 | 10.4 | 2.75* | N.A. | N.A. | N.A. |
| Immediate Visual Memory | ⩾ 13.85 | 19.6 | 22 | 18.75 | 18.75 | N.A. |
| Rey Auditory Verbal Learning Test (RAVLT) | | | | | | |
| Immediate | ⩾ 28.53 | 42.8 | 29.65 | 30.65 | 20.65* | N.A. |
| Delayed | ⩾ 4.69 | 10.6 | 8.85 | 4.85 | 6.3 | N.A. |
| Constructional apraxia | ⩾ 7.18 | 10.1 | 10.2 | 10.2 | 8.2 | N.A. |
| Laiacona semantic memory test | | | | | | |
| Visual naming | ⩾ 61 | – | 73/80 | 68/80 | – | – |
| Semantic questionnaire | ⩾ 447 | – | 461/480 | – | – | – |
| Semantic Fluency | ⩾ 25 | 35 | 10.5* | 13* | 9* | N.A. |
| Boston naming test | ⩾ 16 | 19 | 16* | 15* | 13* | N.A. |
| Digit Span | ⩾ 3.75 | 4.5 | 4.5 | 3.5* | 1.5* | N.A. |
| Visuo-Spatial Span – Corsi Test | ⩾ 3.50 | 5 | 5.12 | 4.12 | 4.12 | N.A. |
| Attentive Matrices | ⩾ 31 | – | – | 18.12* | 18.12* | N.A. |
| Object decision test – BORB subtest 10 Easy A | ⩾ 24 | 28 | 28 | – | – | – |
| Associative match task – BORB subtest 12 | ⩾ 22 | 29 | 29 | – | – | – |
| Stroop test | | | | | | |
| Interference time | ⩽ 36.91 | 69.65* | 76.7* | 37.2* | 59.7* | N.A. |
| Interference errors | ⩽ 4.23 | 2.25 | 0 | 0 | 0.5 | N.A. |
| Wisconsin Card Sorting Test | | | | | | |
| Categories | ⩾ 3 | 2* | 2* | 2* | 1* | N.A. |
| Perseverations | ⩽ 6.40 | 5.75* | 13.75* | 11.75* | 11.75* | N.A. |
| Test for aphasia (Aachener Aphasie Test) | | | | | | |
| Spontaneous speech | | | | | | |
| Communicative Behavior | 4 | 4 | 3 | 2 | 0 | |
| Prosody | 5 | 5 | 5 | 5 | 5 | |
| Automatic language | 5 | 5 | 4 | 4 | 3 | |
| Semantic Structure | 4 | 4 | 4 | 3 | 1 | |
| Phonemic Structure | 5 | 5 | 4 | 3 | 1 | |
| Syntactic Structure | 5 | 5 | 4 | 3 | 2 | |
| Token test | ⩽ 6 | 5 | – | – | – | N.A. |
| Repetition | ⩾ 144 | 144 | 129* | 119* | 114* | N.A. |
| Naming | ⩾ 106 | 101* | 104* | 102* | 89* | N.A. |
| Written language | ⩾ 82 | 88 | 85 | 83 | 82 | N.A. |
| Comprehension | ⩾ 107 | 112 | 113 | 112 | 110 | N.A. |
| Hospital Anxiety and Depression Scale | | | | | | |
| Anxiety | ⩽ 10 | 10 | 8 | 8 | 8 | N.A. |
| Depression | ⩽ 10 | 16* | 8 | 8 | 13* | N.A. |

Legend: N.A. Not Applicable due to illness severity; *pathological scores; – not administered.

like: *"I asked, I looked for, towards and so, co, here around, yes, there is people, it is a hundred"*; in response to the question "When were you born?", patient replies: *"Born? Pento, bande bande and go. That I don't say, to say, danno, I have some, so doctor"*; in response to "Goodbye", patient said: *"Goodbye, ah there will be... no, I saw a porsa, there is a land, I know that migenda vincite avò so so"*. An example of spontaneous speech was: "*rasapele ripata ripata, labela,* debelito, eh, mitota bolu". (Underlined words are non-existent Italian words). The language was totally unintelligible, with fluent jargon characterised by mainly non-existing words and real words put together in meaningless sequences as a mixed jargon [9,10].

The three-dimensional MRI showed an increased atrophy in the left temporo-parietal lobe, in the absence of vascular lesions.

A t-test comparison between the $^{18}$F-FDG PET of the patient and those of a group of ten healthy controls (mean age 68.36, SD 8.59; mean education 9.55,

Table 2
Brain regions of decreased hypo-metabolism in the patient

| Brain area | BA | L/R | Number of voxels in cluster | Cluster-level p-value (corrected) | Z value at local maximum | Talairach coordinates | | |
|---|---|---|---|---|---|---|---|---|
| | | | | | | x | y | z |
| Superior temporal gyrus | 22 | L | 2903 | 0.000 | 5.11 | −59 | −44 | 15 |
| | 22 | R | 253 | | 4.56 | 61 | −55 | 19 |
| | 39 | R | 253 | | 3.91 | 55 | −61 | 18 |
| Angular gyrus | 39 | L | 2903 | 0.000 | 4.98 | −46 | −61 | 33 |
| Middle Temporal gyrus | 21 | L | 2903 | 0.000 | 4.47 | −55 | −56 | 5 |
| Posterior cingulate | 31 | L | 948 | 0.000 | 4.49 | −6 | −57 | 21 |
| Precuneus | 7 | L | 948 | 0.000 | 4.10 | −4 | −62 | 42 |
| Inferior frontal gyrus | 45 | R | 126 | 0.012 | 4.14 | 61 | 24 | 10 |
| | 44 | R | 126 | | 3.74 | 53 | 16 | 14 |
| | 44 | L | 245 | | 3.98 | −51 | 7 | 20 |
| Middle frontal gyrus | 6 | L | 245 | 0.000 | 3.25 | −51 | 4 | 37 |
| Caudate | | L | 165 | 0.002 | 3.73 | −16 | 5 | 16 |

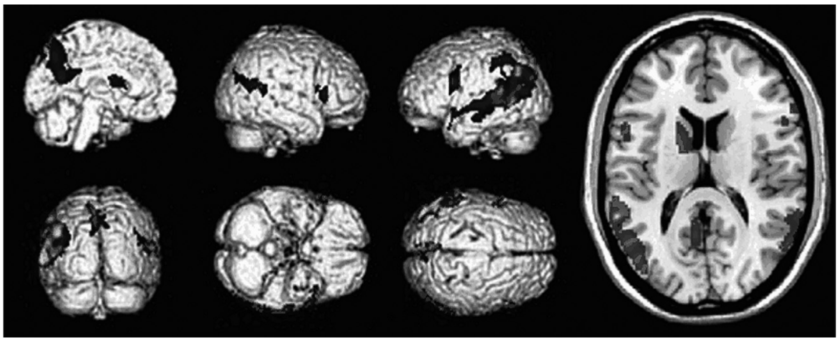

Fig. 1. Areas of hypometabolism revealed by FDG-PET in the patient compared to healthy controls.

SD 4.48) was computed using SPM5. Education was entered as covariate variable in the analysis. Areas of significant hypo-metabolism were found mainly in the left hemisphere in the angular (BA 39) and middle temporal (BA 21) gyri, precuneus (BA 7), posterior cingulate (BA 31), middle frontal gyrus (BA 6), caudate, and bilaterally in the superior temporal (BA 22) and inferior frontal (BA 44) gyri, ($p < 0.001$), (see Table 2 and Fig. 1).

The CSF analysis revealed a significant decrease of ß-amyloid (203, cut off > 600 pg/mL), a slight increase of total Tau protein (394, cut off < 300 pg/mL) and normal value of phosphorylated Tau (value 42, cut off < 60 pg/mL). APOE genotype was $\varepsilon 3/\varepsilon 3$.

## 3. Discussion

The language disorder profile of the patient at onset was characterised by anomic pauses, and progressed within ten years into a severe form of fluent, unintelligible speech including a severe deficit in repetition, corresponding to the characteristics of mixed, semantic and neologistic jargonaphasia, associated with severe comprehension deficits. The recent $^{18}$F-FDG PET exam showed a significant hypo-metabolism in the same regions evidenced by at onset SPECT scan, but with an increase in damage extension in left fronto-temporo-parietal regions, extending also to the controlateral superior temporal and inferior frontal cortex (BA 44 and BA 22, respectively Broca's and Wernicke's areas).

From the neurolinguistic standpoint, the cause of jargon is a matter of debate. It is often considered as a consequence of defective monitoring of language production, due to defective auditory comprehension, associated with a severe impairment of phonological encoding [11]. Neologisms may be the production of a phonological sequence generator to fill "gaps" in lexical retrieval [12,13]. In vascular aphasia, jargon is typically observed in the context of Wernicke's aphasia, associated with damage in the posterior-superior temporal cortex. The results of some neuropathological studies, as well as of neuroimaging investigations, suggest that extension of damage towards the temporal and parietal cortex is responsible for jargon occurrence. More specifically, parietal vs. temporal extension may be associated with, respectively, semantic and neologistic jargon [14].

The prominent involvement of the temporo-parietal region observed in our patient, as well as in the cases reported by Demeracourt [8] and Rohrer [9], are in agreement with the data from vascular aphasia.

Why is progressive jargonaphasia rare, and how is it related to the three main clinical varieties of PPA? A prominent involvement of the left temporo-parietal regions is typical of the logopenic/phonological variety, which is often due to Alzheimer's disease. Jargonaphasia may thus represent a relatively unusual evolution of this syndrome. The evolution may be late, such as in the present case, or relatively early, as in Rohrer's case 1. The possibility that the responsible pathology may be AD is strengthened by the observation of the low CSF ß-amyloid level and high level of total Tau found in our patient. The patient was not $\varepsilon 4$ carrier, and this is consistent with the atypical forms of AD [15].

The present clinical observation provides further evidence for the heterogeneity of clinical presentations of PPA, which reflects underlying differences in the topographical pattern of pathological involvement. This variability may be particularly extensive in the case of the logopenic/phonological variant, frequently associated with atypical patterns of AD neuropathology.

## Acknowledgments

The authors thank the patient and the patient's family for the cooperation. This study was supported by a grant from the Fondazione Cassa di Risparmio of Parma and Piacenza to PC.

## References

[1] M.M. Mesulam, Primary progressive aphasia, *Ann Neurol* **49** (2001), 425–432.

[2] D.G. Clark, A. Charuvastra, B.L. Miller et al., Fluent versus non fluent primary progressive aphasia: A comparison of clinical and functional neuroimaging features, *Brain Lang* **94** (2005), 54–60.

[3] M.L. Gorno-Tempini, N.F. Dronkers, K.P. Rankin et al., Cognition and Anatomy in Three Variants of Primary Progressive Aphasia, *Ann Neurol* **55** (2004), 335–346.

[4] M.L. Gorno-Tempini, S.M. Brambati, V. Ginex et al., The logopenic/phonological variant of primary progressive aphasia, *Neurology* **71** (2008), 1227–1234.

[5] M.L. Gorno-Tempini, A.E. Hillis, S. Weintraub et al., Classification of primary progressive aphasia and its variants, *Neurology* **76** (2011), 1006–1014.

[6] P. Ostberg, N. Bogdanovic, S.E. Fernaeus et al., Jargonagraphia in a case of frontotemporal dementia, *Brain Lang* **79** (2001), 333–339.

[7] N.L. Graham, K. Patterson and J.R. Hodges, The emergence of jargon in progressive fluent dysgraphia: The widening gap between target and response, *Cogn Neuropsychol* **18** (2001), 343–361.

[8] V. Deramecourt, F. Lebert, B. Debachy et al., Prediction of pathology in primary progressive language and speech disorders, *Neurology* **74** (2010), 42–49.

[9] J.D. Rohrer, M.N. Rossor and J.D. Warren, Neologistic jargon aphasia and agraphia in primary progressive aphasia, *J Neurol Sci* **277** (2009), 155–159.

[10] B. Butterworth, Jargon aphasia: processes and strategies, in: S. Newman and R. Epstein, eds, Current perspectives in dysphasia. Edinburgh, UK: Churchill Livingstone, 1985, pp. 61–96.

[11] A. Kertesz and D.F. Benson, Neologistic jargon: a clinicopathological study, *Cortex* **6** (1970), 362–386.

[12] H.W. Buckingham and A. Kertesz, Neologistic jargon aplasia. Amsterdam: Swets and Zeitlinger, 1976.

[13] B. Butterworth, Hesitation and the Production of Verbal Paraphasias and Neologisms in Jargon Aphasia, *Brain Lang* **8** (1979), 133–161.

[14] A. Kertesz, Neuropsychological evaluation of language, *J Clin Neurophysiol* **11** (1994), 205–215.

[15] W.M. van der Flier, Y.A. Pijnenburg, N.C. Fox et al., Early-onset versus late-onset Alzheimer's disease: the case of the missing APOE $\varepsilon 4$ allele, *Lancet Neurol* **10** (2011), 280–288.
